# Spin dynamics of the block orbital-selective Mott phase

**DOI:** 10.1038/s41467-018-06181-6

**Published:** 2018-09-13

**Authors:** J. Herbrych, N. Kaushal, A. Nocera, G. Alvarez, A. Moreo, E. Dagotto

**Affiliations:** 1 Department of Physics and Astronomy, University of Tennessee, Knoxville, TN 37996 USA; 2 Materials Science and Technology Division, Oak Ridge National Laboratory, Oak Ridge, TN 37831 USA; 3 Computational Sciences and Engineering Division and Center for Nanophase Materials Sciences, Oak Ridge National Laboratory, Oak Ridge, TN 37831 USA

## Abstract

Iron-based superconductors display a variety of magnetic phases originating in the competition between electronic, orbital, and spin degrees of freedom. Previous theoretical investigations of the multi-orbital Hubbard model in one-dimension revealed the existence of an orbital-selective Mott phase (OSMP) with block spin order. Recent inelastic neutron scattering (INS) experiments on the BaFe_2_Se_3_ ladder compound confirmed the relevance of the block-OSMP. Moreover, the powder INS spectrum revealed an unexpected structure, containing both low-energy acoustic and high-energy optical modes. Here we present the theoretical prediction for the dynamical spin structure factor within a block-OSMP regime using the density-matrix renormalization-group method. In agreement with experiments, we find two dominant features: low-energy dispersive and high-energy dispersionless modes. We argue that the former represents the spin-wave-like dynamics of the block ferromagnetic islands, while the latter is attributed to a novel type of local on-site spin excitations controlled by the Hund coupling.

## Abstract (web summary)

Exploring the orbital-selective Mott phase (OSMP) addresses the central issue of electron correlations in the iron-based superconductors. Here the authors theoretically study the dynamical spin structure factor in the block-OSMP regime and unveil momentum dependent characteristics for different spin excitation modes.

## Introduction

Inelastic neutron scattering (INS) measurements are crucial for the study of quantum magnetism in condensed matter physics. This powerful experimental technique provides detailed information of momentum and energy resolved spin excitations. The importance of INS studies is best illustrated in the case of high critical temperature superconductors. Shortly after the discovery of the copper-oxide compounds it became evident that the standard BCS theory of the electron–phonon coupling could not explain the experimental findings. Simultaneously, INS results showed that superconductivity appears in close proximity to the antiferromagnetic (AFM) ordering of *S* = 1/2 Cu^2+^ moments providing robust evidence that the new pairing mechanism is based on spin fluctuations^1–4^.

The discovery of iron-based superconductors (FeSC) added an extra complication to this “simple” picture. Although the phase diagrams of Cu-based and Fe-based materials are qualitatively similar^5^, there are important conceptual differences. The most significant are in the minimal models that describe the materials^6,7^. While cuprates have a single Fermi surface (FS), the iron-based compounds have a complicated FS with electron and hole pockets originating in the five 3*d* orbitals of iron. As a consequence, the FeSC have to be described by means of multi-orbital Hubbard models, involving not only a standard Hubbard *U* repulsion but also a Hund coupling. The competition between electronic, orbital, and spin degrees of freedom can lead to many exotic magnetic phases^8–13^.

Past experience in cuprates showed that the analysis of lower dimensional systems, such as chains and ladders, can provide useful information to better contrast theory with experiments^14^. One reason is that theoretical many-body calculations based on model Hamiltonians can be accurately performed in one-dimension, particularly numerically. For this reason, it was exciting when a one-dimensional family of compounds containing two-leg ladders was unveiled also in the iron-superconductors context. Specifically, we refer to the low-dimensional FeSC in the 123 family, *A*Fe_2_*X*_3_, where *A* are alkali metals *A* = K, Ba, Rb, Cs, and *X* are chalcogenides *X* = S, Se. These compounds are build of double chains (i.e., they are ladders) of edge sharing Fe*X*_4_ tetrahedra^15^. Recently, a superconducting state was identified under pressure for BaFe_2_S_3_^16,17^ and BaFe_2_Se_3_^18,19^. The pressure-dependent phase diagram of these materials resembles that of copper-oxide ladders, e.g., the telephone number compound Sr_14−*x*_Ca_*x*_Cu_24_O_41_^20^. Similar to their copper-oxide counterparts, the iron-123 family is insulating at ambient pressure. This behavior is unusual since, unlike the cuprates, the parent compounds of FeSC are typically bad metals. In addition, it was argued that orbital-selective Mott physics (OSMP)^21^ is consistent with results for BaFe_2_Se_3_^22–25^. Within such a phase, itinerant and localized conduction electrons coexist.

It should be remarked that INS experiments on 123 materials have been performed up to now only on powder samples and, as a consequence, detailed data of the momentum dependence of the spin excitations over the whole Brillouin zone is not yet available. Nevertheless, the static (*π*, 0) stripe AFM order—with ferromagnetic rungs and antiferromagnetic legs—was identified for BaFe_2_S_3_^26^, RbFe_2_Se_3_^27^, CsFe_2_Se_3_^28,29^, and also for KFe_2_S_3_^22^. However, in the special case of BaFe_2_Se_3_ remarkably an exotic block magnetism was found^19,22,25,30,31^ involving antiferromagnetically coupled ferromagnetic islands made of 2 × 2 iron clusters. This unusual magnetic state was also observed in the vicinity of superconductivity^32–34^ in two-dimensional (2D) materials with $\sqrt 5\, \times \sqrt 5$ ordered iron vacancies, such as Rb_0.89_Fe_1.58_Se_2_^35^ and K_0.8_Fe_1.6_Se_2_^36–38^. In addition, for BaFe_2_Se_3_^25^, BaFe_2_S_3_^26^, and RbFe_2_Se_3_^27^ the INS revealed the existence of low-energy acoustic and high-energy optical modes separated by an energy gap. It is important to remark that the generic features of the INS spectra of the aforementioned compounds are similar, but the physical origin of the acoustic modes can differ significantly—these modes reflect on the long-distance properties of the magnetic order in the system. Moreover, the origin and characteristics of the optical modes, that are induced by short-distance properties, have not been clarified so far.

In this work, we will address the spin dynamical properties of the exotic block magnetic state found in BaFe_2_Se_3_. The static (time independent) properties of this phase were previously qualitatively studied in ref.^23^ via a three-orbital Hubbard model in one-dimension (1D) that unveiled an OSMP regime. Here, we will use the same Hamiltonian to investigate the momentum and energy resolved spin dynamics. To test the general features of our findings, we present also results obtained in a quasi-1D ladder geometry. In agreement with experimental findings, we have observed two distinct modes of spin excitations: a low-energy dispersive mode and high-energy dispersionless optical modes. The low-energy acoustic mode reveals the frustrated nature of the block magnetism which can be described by a spin *J*_1_–*J*_2_ Hamiltonian. On the other hand, we argue that the optical mode is controlled by local orbital physics and it cannot be properly captured by a Heisenberg-like model. The main features of our analysis are simple and generic and should characterize any multi-orbital model as long as its ground state is in a magnetic block phase.

## Results

### Model and observables

**Fig. 1:**
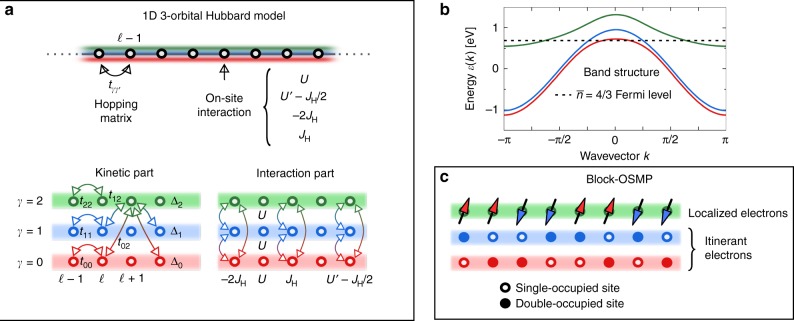
Schematic representation of the Hamiltonian. **a** Three-orbital Hubbard model on a one-dimensional lattice geometry (see text for details). **b** Band structure of Hamiltonian Eq. (1). Note that due to the hybridization $t_{\gamma \gamma \prime } \ne 0$ for *γ* ≠ *γ*′, the band numbers do not correspond directly to the orbital numbers. **c** Schematic representation of the block orbital-selective Mott phase. The pattern of single and double occupied sites in the itinerant electrons is meant to be random, representing pictorially the not-localized nature of those orbitals

We will focus on a specific three-orbital Hubbard model on a one-dimensional lattice, but our conclusions are generic for a broad group of models and materials in the OSMP magnetic block-phase regime. As mentioned before, the model chosen was previously studied with regards to its time-independent properties, and it is known that it displays an OSMP regime in the ground state^23^. The kinetic part of the Hamiltonian, *H*_kin_, is defined as:

1 $$H_{{\mathrm{kin}}} = - \mathop {\sum}\limits_{\ell ,\sigma ,\gamma ,\gamma \prime } t_{\gamma \gamma \prime }\left( {c_{\ell ,\gamma ,\sigma }^\dagger c_{\ell + 1,\gamma \prime ,\sigma } + {\mathrm{H}}{\mathrm{.c}}{\mathrm{.}}} \right) + \mathop {\sum}\limits_{\ell ,\gamma ,\sigma } {\kern 1pt} {\mathrm{\Delta }}_\gamma n_{\ell ,\gamma ,\sigma },$$

where $c_{\ell ,\gamma ,\sigma }^\dagger$ creates an electron with spin *σ* = {↑, ↓} at orbital *γ* = {0, 1, 2} and site $\ell = \{ 1, \ldots ,L\}$ of a 1D chain. $n_{\ell ,\gamma ,\sigma } = c_{\ell ,\gamma ,\sigma }^\dagger c_{\ell ,\sigma ,\gamma }$ is the local $(\ell ,\gamma )$ electron density with spin *σ*. Note that another common labeling of these orbitals could be based on the canonical *t*_2*g*_ manifold, i.e., {*yz*, *xz*, *xy*}, respectively. $t_{\gamma \gamma \prime }$ denotes a symmetric hopping amplitude matrix defined in the orbital space *γ*: *t*_00_ = *t*_11_ = −0.5, *t*_22_ = −0.15, *t*_02_ = *t*_12_ = 0.1, and *t*_01_ = 0, all in eV units (Fig. 1a displays a schematic representation of the Hamiltonian). The crystal-field splitting is set to Δ_0_ = −0_1_, Δ_1_ = 0, and Δ_2_ = 0.8, also in eV units. The total kinetic-energy bandwidth is *W* = 2.45 eV. These phenomenological values of parameters were chosen before^23^ to reproduce qualitatively the band structure properties of higher dimensional selenides at an electronic density $\bar n = 4{\mathrm{/}}3$ per orbital, namely an electron-like poc*k*et at *k* = 0 and hole-like poc*k*ets at *k* = ±*π* (see Fig. 1b, and also ref.^39^ and references therein). It should be pointed out that the existence of an OSMP highlights the striking orbital sensitivity on electron correlations in multi-orbital Hubbard models, and its presence is not limited to our use of 1D geometries nor to our choice of $t_{\gamma \gamma \prime }$ hoppings. For example, the OSMP was proven to be relevant^40^ for 2D alkaline iron selenides as well, with and without $\sqrt 5\, \times \sqrt 5$ ordered vacancies. We wish to emphasize that our predictions primarily depend on the existence of an OSMP magnetic block-phase state, rather than on the details of the Hamiltonian that leads to its stabilization. In this context, we believe that our results are universal for iron-based superconductors. To support this claim, we will present calculations for several models showing that all the many reported results lead essentially to the same qualitative conclusions.

The interaction portion of the Hamiltonian *H*_int_ is given by

2 $$\begin{array}{*{20}{l}} {H_{{\mathrm{int}}}} \hfill & = \hfill & {U\mathop {\sum}\limits_{\ell ,\gamma } {\kern 1pt} n_{\ell ,\gamma , \uparrow }n_{\ell ,\gamma , \downarrow } + (U\prime - J_{\mathrm{H}}{\mathrm{/}}2)\mathop {\sum}\limits_{\ell ,\gamma < \gamma \prime } {\kern 1pt} n_{\ell ,\gamma }n_{\ell ,\gamma \prime }} \hfill \\ {} \hfill & {} \hfill & { - 2J_{\mathrm{H}}\mathop {\sum}\limits_{\ell ,\gamma < \gamma \prime } {\kern 1pt} S_{\ell ,\gamma }S_{\ell ,\gamma \prime } + J_{\mathrm{H}}\mathop {\sum}\limits_{\ell ,\gamma < \gamma \prime } \left( {P_{\ell ,\gamma }^ + P_{\ell ,\gamma \prime } + {\mathrm{H}}{\mathrm{.c}}{\mathrm{.}}} \right),} \hfill \end{array}$$

where $n_{\ell ,\gamma } = \mathop {\sum}\nolimits_\sigma {\kern 1pt} n_{\ell ,\gamma ,\sigma }$, the local spin $(\ell ,\gamma )$ is $S_{\ell ,\gamma }$ = $(1{\mathrm{/}}2)\mathop {\sum}\nolimits_{a,b} {\kern 1pt} c_{\ell ,\gamma ,a}^\dagger \sigma ^{ab}c_{\ell ,\gamma ,b}$ (with *σ*^*ab*^ as a Pauli spin matrices), and $P_{\ell ,\gamma }$ = $c_{\ell , \uparrow ,\gamma }c_{\ell , \downarrow ,\gamma }$ is the pair-hopping. We will consider an SU(2) symmetric system, i.e., *U*′ = *U* − 2*J*_H_, where *U* stands for the on-site same-orbital repulsive Hubbard interaction. Finally, we set the Hund coupling to *J*_H_ = *U*/4, a value widely used before and considered to be realistic for Fe-based materials^41,42^. We refer the interested reader to refs.^23,43,44^ for details of the *J*_H_–*U* phase diagram of the above Hamiltonian. Here, if not stated differently, we will use *U*/*W* = 0.8 where previous studies found^23^ a block-OSMP, i.e., antiferromagnetically (AFM) coupled ferromagnetic (FM) blocks (magnetic unit cells), ↑↑↓↓↑↑↓↓, in the localized orbital *γ* = 2 (see Fig. 1c). Note that the block order is usually studied in the context of Heisenberg-like spin Hamiltonians (such as dimerized^25,35^ or *J*_1_–*J*_2_ models^45^). Here, the block phase is a consequence of nontrivial electronic correlations within the OSMP phase. Since the latter is a feature of multi-orbital systems that cannot be analyzed using purely spin systems, we believe that our setup is more suitable for the study of iron-based materials.

In this work, we will investigate the zero-temperature frequency *ω*-dependent spin structure factor (SSF) *S*(*q*, *ω*), defined as the Fourier transform of the real-space total (on-site, $S_\ell = \mathop {\sum}\nolimits_\gamma {\kern 1pt} S_{\ell ,\gamma }$) spin correlation functions (see Methods). Furthermore, we will study the contributions from the individual orbitals to the total SSF, i.e., $S_{\gamma \gamma \prime }(q,\omega )$. *γ* = *γ*′ denotes the spin fluctuations within each of the orbitals, while *γ* ≠ *γ*′ are spin fluctuations between different orbitals. As a consequence $S(q,\omega )$ = $\mathop {\sum}\nolimits_\gamma {\kern 1pt} S_{\gamma \gamma }(q,\omega ) + \mathop {\sum}\nolimits_{\gamma \ne \gamma \prime } {\kern 1pt} S_{\gamma \gamma \prime }(q,\omega )$. From the experimental perspective, only the total SSF has a meaning^46^ because neutrons couple to electrons in all orbitals in neutron scattering experiments. However, the theoretical investigations of orbital-resolved SSF can provide further insight into the OSMP physics.

The Hamiltonians are diagonalized via the DMRG method, where the dynamical correlation functions are obtained with the help of dynamical DMRG techniques (see Methods and Supplementary Note 1 for details of the numerical simulations).

### Dynamical spin structure factor

**Fig. 2:**
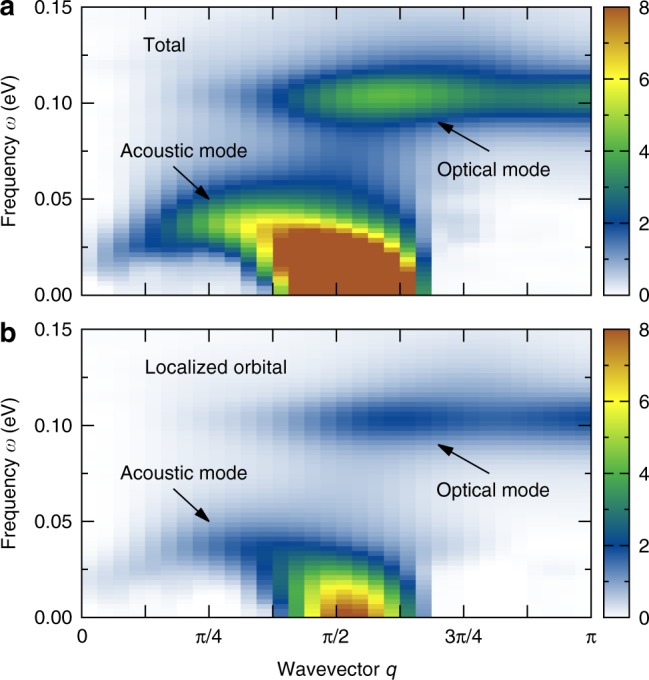
Dynamical spin structure factor (SSF). **a** Total SSF, *S*(*q*, *ω*), and **b** SSF of the localized orbital, *S*_22_(*q*, *ω*). Both results exhibit a low-energy acoustic and a high-energy optical modes. Note that the spectral weight of the localized orbital, *S*_22_, constitutes ~50% of the total SSF weight. The results were obtained using a dynamical DMRG method with parameters *L* = 16 (48 orbitals), *M* = 800, *δω* = 0.005 eV, and *η*/*δω* = 2

In Fig. 2, we present one of the main results of our effort: the frequency-momentum dependence of the dynamical SSF in the block-OSMP phase (i.e., at *U*/*W* = 0.8). Figure 2a depicts the total SSF, *S*(*q*, *ω*), while Fig. 2b shows only the contribution from the localized orbital, *S*_22_(*q*, *ω*). Several conclusions can be obtained directly from the presented results: (i) A robust contribution to the total SSF arises from the localized orbital. Moreover, all the qualitative features of *S*(*q*, *ω*) are already present in *S*_22_(*q*, *ω*). In fact, *S*(*q*, *ω*) and *S*_22_(*q*, *ω*) become almost indistinguishable if normalized by the local magnetic moment squared (i.e., *S*^2^ = 3/4 for the *S* = 1/2 localized electron, and *S*^2^ = 2 for the total moment^23^). (ii) The energy range for the spin dynamics is much smaller when compared with the energy bandwidth *W* = 2.45 eV of the Hamiltonian. (iii) Clearly the dynamical SSF has two distinct modes: a low-frequency, *ω* ≲ *ω*_*c*_ = 0.08 eV, dispersive (acoustic) band and a high-frequency, *ω* ~ 0.11 eV, dispersionless (optical) band. Similar results were previously reported experimentally in INS investigations of BaFe_2_Se_3_^25^ (with 2 × 2 FM blocks), BaFe_2_S_3_^26^, and RbFe_2_Se_3_^27^ (with 2 × 1 FM blocks). The different types of blocks in the INS investigations, and the similarity of results between neutrons and our calculations, suggest that our results apply to a broad variety of iron chalcogenides. Moreover, the INS measurements where performed on powder samples and, as a consequence, no detailed analysis of the spin excitations over all crystal momenta *q* (over the whole Brillouin zone) have been reported. In this respect, our results define clear theoretical predictions on what future single-crystal experiments should display.

**Fig. 3:**
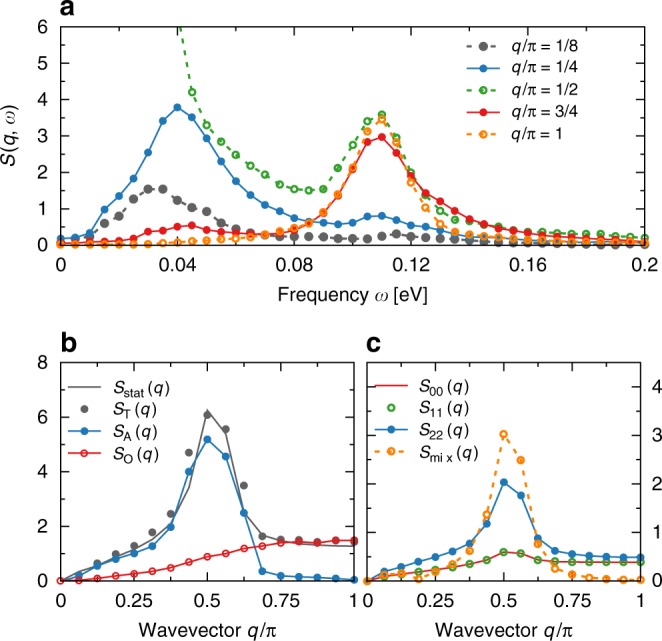
Frequency and momentum dependence. **a** Finite momentum cuts, *q*/*π* = 1/8, 1/4, 1/2, 3/4, 1, of the dynamical SSF. **b** Total static SSF obtained as an expectation value in the $\left| {{\mathrm{GS}}} \right\rangle$, *S*_stat_(*q*) (black line), and also via the *ω* integration of the dynamical SSF, *S*_T_(*q*) (black points). In the same panel, we present also the contributions to the static SSF from acoustic and optical modes, *S*_A_(*q*) and *S*_O_(*q*), respectively. **c** Contribution to the static SSF $S_{\gamma \gamma \prime }(q)$: *γ* = *γ*′ represents the SSF component for each of the orbitals, while $S_{{\mathrm{mix}}}(q)$ = $\mathop {\sum}\nolimits_{\gamma \ne \gamma \prime } S_{\gamma \gamma \prime }(q)$ represents the sum of the inter-orbital contributions. The system parameters are the same as in Fig. 2

In Fig. 3a, we present the *ω* dependence of the total SSF at special values of the momenta *q*. It is evident that the acoustic mode is strongly momentum dependent in the range 0 < *q*/*π* ≲ 1/2, while it reduces drastically its intensity for *q*/*π* > 1/2. To understand these results, we can reanalyze the SSF spectrum using two-sites as a rigid block, namely creating an effective magnetic unit cell of FM blocks with momentum $\tilde q$. The acoustic mode as a function of $\tilde q$ then is located between $0 \, < \, \tilde q \, < \, \pi$, resembling a gapless continuum of spin excitations. Such an interpretation is consistent with “collective” spin waves based on FM blocks. On the other hand, the high-energy optical contribution is *q*-independent for *q*/*π* ≳ 1/2, with vanishing intensity in the *q* → 0 limit. As discussed later, this mode can be associated to local (on-site) spin excitations affecting the Coulomb potential portion of the Hamiltonian, independently of the dimensionality of the system. The *q*-dependence of both modes is also clearly visible in the static SSF obtained from the energy integration of the dynamical SSF, i.e., $S_\alpha (q)$ = $(1{\mathrm{/}}\pi ){\int} {\kern 1pt} {\mathrm{d}}\omega {\kern 1pt} S(q,\omega )$. In Fig. 3b, we present the acoustic (*α* = A) and optical (*α* = O) contribution to the total (*α* = *T*) static SSF, coming from the integration over the frequency ranges 0 < *ω* < *ω*_*c*_, *ω*_*c*_ < *ω* < ∞, and 0 < *ω* < ∞, respectively. From the dynamical SSF spectra, it is evident that *S*_O_(*q*) provides the sole contribution to the total static SSF for momentum 0.75 < *q*/*π* < 1. As a consequence, at least within a block-OSMP state it is remarkable that already in the static SSF one can observe the clear presence of an optical mode, a novel result which is intrinsic of block phases to our knowledge. In the same panel, we also present the total static SSF independently obtained from the expectation value of the ground state (GS), i.e., *S*_stat_(*q*) = 〈GS|*S*_*q*_·*S*_−*q*_$\left| {{\mathrm{GS}}} \right\rangle$, where *S*_*q*_ is the Fourier transform of the $S_\ell$ operators for the same system size *L*. The agreement between *S*_stat_(*q*) and *S*_T_(*q*) serves as nontrivial accuracy test of the dynamical DMRG method, since the former can be obtained with much higher accuracy.

### Orbital contribution

Before addressing the optical and acoustic modes in more detail, we will comment on the orbital *γ* contribution to *S*(*q*, *ω*). As already shown in Fig. 2, the main contribution to the total SSF originates in the localized orbital *γ* = 2. Our results (Fig. 3c) indicate that the spin fluctuations for the itinerant electrons (orbitals *γ* = 0 and *γ* = 1) follow the behavior of the localized orbital. As argued below, this is a consequence of the Hund coupling which aligns ferromagnetically spins at different orbitals. However, the nature of these orbitals is metallic and magnetic moments are not well formed. As a consequence, the spectral weight of the total itinerant contribution (two orbitals) is approximately the same as the localized (one orbital). On the other hand, the inter-orbital SSF $S_{\gamma \ne \gamma \prime }$ have a large contribution only to the acoustic mode, especially near the *q*/*π* = 1/2 point.

### Acoustic mode

**Fig. 4:**
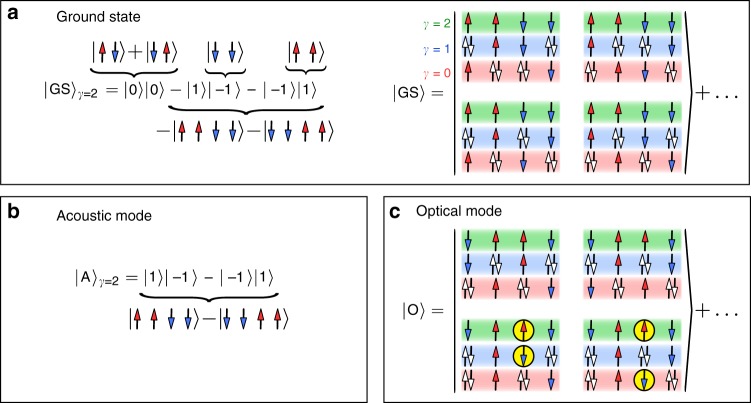
States relevant for the dynamical SSF. Spin configuration in the localized orbital (*γ* = 2) (see text for details) of the **a** $\left| {{\mathrm{GS}}} \right\rangle$ (singlet) and **b** $\left| {\mathrm{A}} \right\rangle$ state (triplet) contributing to the acoustic mode. **c** Schematic representation of particle configuration of all orbitals of the $\left| {{\mathrm{GS}}} \right\rangle$ and optical triplet $\left| {\mathrm{O}} \right\rangle$. Circles represent pairs of antiferromagnetically aligned spins which break the Hund’s rule

Consider now the properties of the acoustic mode. Motivated by the results presented above, with the main contribution to the SSF arising from the localized orbital, we express the eigenstates in terms of the basis states of localized orbital |·〉_*γ*=2_ (see Methods section). Since the electrons are indeed localized with occupation *n*_*γ*=2_ = 1^23^ in the OSMP, in the low-energy portion of the spectrum the basis states with empty and double occupied orbital *γ* = 2 should not be present. Within such a representation the GS of the block-OSMP phase can be identified as a superposition of $\left| { \uparrow \uparrow \downarrow \downarrow } \right\rangle _{\gamma = 2}$ and $\left| { \downarrow \downarrow \uparrow \uparrow } \right\rangle _{\gamma = 2}$ states, which constitutes ~50% of the true GS. One can improve further the qualitative description by investigating a simple toy model. Let us consider two FM coupled *S* = 1/2 spins as one *S* = 1 object, i.e., $\left| {\mathbf{1}} \right\rangle = \left| { \uparrow \uparrow } \right\rangle _{\gamma = 2}$, $\left| { - {\mathbf{1}}} \right\rangle = \left| { \downarrow \downarrow } \right\rangle _{\gamma = 2}$, and $\left| {\mathbf{0}} \right\rangle$ = $1{\mathrm{/}}\sqrt 2 \left( {\left| { \uparrow \downarrow } \right\rangle _{\gamma = 2} + \left| { \uparrow \downarrow } \right\rangle _{\gamma = 2}} \right)$. In this setup, a 4-site *S* = 1/2 system reduces to two antiferromagnetically coupled *S* = 1 spins. The ground state of the latter is simply

3 $$\left| {{\mathrm{GS}}} \right\rangle _{\gamma = 2} = c_a\left| {\mathbf{0}} \right\rangle \left| {\mathbf{0}} \right\rangle - c_b\left( {\left| {\mathbf{1}} \right\rangle \left| { - {\mathbf{1}}} \right\rangle + \left| { - {\mathbf{1}}} \right\rangle \left| {\mathbf{1}} \right\rangle } \right),$$

where $c_a = c_b = 1{\mathrm{/}}\sqrt 3$ (see Fig. 4a for a schematic representation). Note that the above state, in agreement with numerics, is a singlet. The last two terms of Eq. (3) correspond to the “perfect” block order, i.e., $\left| { \uparrow \uparrow \downarrow \downarrow } \right\rangle _{\gamma = 2} + \left| { \downarrow \downarrow \uparrow \uparrow } \right\rangle _{\gamma = 2}$, while the first term depicts the *x*–*y* component of the block order,

4 $$\begin{array}{*{20}{l}} {\left| {\mathbf{0}} \right\rangle \left| {\mathbf{0}} \right\rangle } \hfill & = \hfill & {\frac{1}{2}\left( {\left| { \uparrow \downarrow \uparrow \downarrow } \right\rangle _{\gamma = 2} + \left| { \downarrow \uparrow \downarrow \uparrow } \right\rangle _{\gamma = 2}} \right.} \hfill \\ {} \hfill & {} \hfill & {\left. { + \left| { \uparrow \downarrow \downarrow \uparrow } \right\rangle _{\gamma = 2} + \left| { \downarrow \uparrow \uparrow \downarrow } \right\rangle _{\gamma = 2}} \right).} \hfill \end{array}$$

Our *L* = 4 Lanczos investigation of the full Hamiltonian (1) and (2) indicates that such a state has coefficients equal to $\widetilde c_a^{\, 2} \simeq 1{\mathrm{/}}6$ and $\widetilde c_b^{\,2} \simeq 1{\mathrm{/}}4$, which yields now a better overlap, ~70%, with the true GS. Finally, the first excited state—contributing to the acoustic mode—can be identified as a triplet of the form $\left| {\mathrm{A}} \right\rangle _{\gamma = 2}$ = $\widetilde c_A\left( {\left| { \uparrow \uparrow \downarrow \downarrow } \right\rangle _{\gamma = 2} - \left| { \downarrow \downarrow \uparrow \uparrow } \right\rangle _{\gamma = 2}} \right)$, where $\widetilde c_A^{\,2} \simeq 4{\mathrm{/}}9$ (Fig. 4b). This large overlap of $\left| {\mathrm{A}} \right\rangle _{\gamma = 2}$ with the full solution is also captured by the toy model since |**1**〉|−**1**〉 − |−**1**〉|**1**〉 is one of the first excitations in our two-site *S* = 1 problem. Note that the above description of the $\left| {{\mathrm{GS}}} \right\rangle _{\gamma = 2}$ $\left( {\left| {\mathrm{A}} \right\rangle _{\gamma = 2}} \right)$ as a spin singlet (triplet) is not obvious from the signs of the localized orbital basis representation. While the above states capture the essence of the problem, the itinerant orbitals have to be included in the description to account for the true nature of the singlet-triplet excitation.

**Fig. 5:**
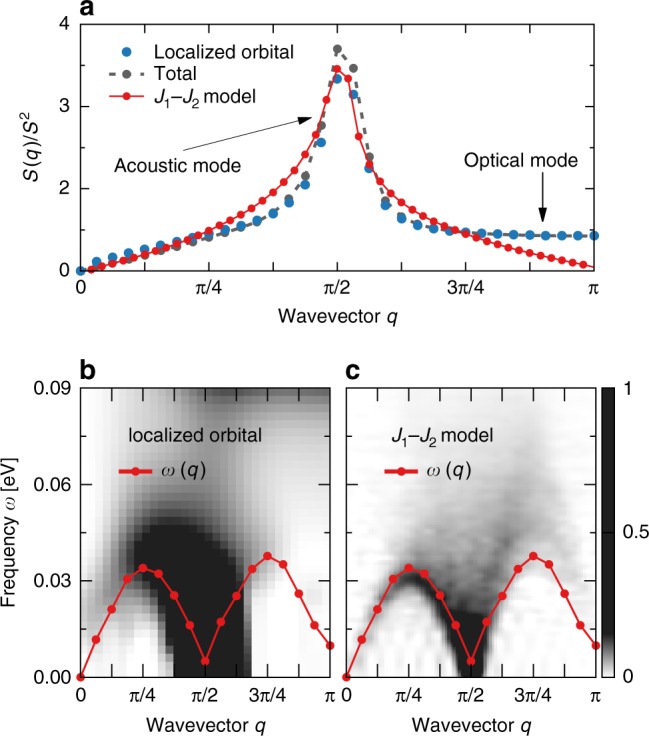
Comparison with the *J*_1_–*J*_2_ model. **a** Comparison of the static SSF *S*(*q*) corresponding to the multi-orbital system vs results for the *J*_1_–*J*_2_ model with *J*_2_/|*J*_1_| = 1, calculated for *L* = 32 and *L* = 48, respectively. *S*(*q*) is normalized by the local magnetic moment squared *S*^2^ = *S*(*S* + 1), where *S*^2^ = 3/4 for the localized orbital and the *J*_1_–*J*_2_ result, and *S*^2^ = 2^23^ for the total SSF. **b** Acoustic mode of the dynamical SSF within the block-OSMP phase (the same results as in Fig. 2b) compared against the dispersion relation *ω*(*q*) of the *J*_1_–*J*_2_ model with |*J*_1_| = *J*_2_ = 0.6*J*_eff_, where the effective spin-exchange energy scale is set to $J_{{\mathrm{eff}}} = 4{\kern 1pt} t_{22}^2{\mathrm{/}}U$. The latter is obtained with the help of Lanczos diagonalization on a chain of 32 sites with periodic boundary conditions. **c** Dynamical SSF of the *J*_1_–*J*_2_ model with |*J*_1_| = *J*_2_ = 0.6*J*_eff_ as calculated using DMRG for a *L* = 48 chain

Although simplified, descriptions such as those above of the low-energy spectrum can yield nontrivial consequences. A similar ground state to our $\left| {{\mathrm{GS}}} \right\rangle _{\gamma = 2}$ with *π*/2 pitch angle was previously observed in the frustrated ferromagnetic *S* = 1/2 *J*_1_–*J*_2_ Heisenberg model with ferromagnetic *J*_1_ and antiferromagnetic *J*_2_^45,47–50^. In Fig. 5a, we present a comparison of the multi-orbital system Eqs. (1) and (2) SSF vs *J*_1_–*J*_2_ results obtained for *J*_2_/|*J*_1_| = 1. Within the latter, the dynamical SSF yields a continuum of excitations with maximum intensity at *q*/*π* = 1/2 and vanishing intensity in the *q*/*π* → 1 limit. In fact, the dynamical SSF of the *J*_1_–*J*_2_ model is very similar to the acoustic mode found in our multi-orbital system, i.e., compare panels (b) and (c) of Fig. 5. To strengthen this argument, in Fig. 5b and c, we present the dynamical SSF factor plotted against the quantum dispersion relation of the *J*_1_–*J*_2_ model *ω*(*q*) = $\epsilon _q$ − $\epsilon _{{\mathrm{GS}}}$, where $\epsilon _q$ is the energy of the lowest eigenstate at a given *q*. To match the energy scales, we set |*J*_1_| = *J*_2_ = 0.6*J*_eff_, where $J_{{\mathrm{eff}}} = 4{\kern 1pt} t_{22}^2{\mathrm{/}}U$ is the natural superexchange scale within the localized orbital, as a crude approximation. As clearly shown in Fig. 5b, *ω*(*q*) quantitatively captures the main features of the acoustic portion of the spectrum.

We remark that the present comparison with the *J*_1_–*J*_2_ model is at a phenomenological level, since this effective description of the lowest mode of the spin dynamics was not rigorously derived from our multi-orbital Hamiltonian Eqs. (1) and (2). The acoustic mode reflects the frustrated nature of the magnetism within the block-OSMP phase. Also, the *J*_1_–*J*_2_ model may be relevant in a wide range of interaction *U* within the OSMP phase, beyond the block ordering region 0.4 ≲ *U*/*W* ≲ 1.5. For example, previous results showed that in the range 1.5 ≲ *U*/*W* ≲ 20, the system is in a ferromagnetic-OSMP^23^, where the spins within the localized orbital *γ* = 2 have ferromagnetic ordering. Clearly, a *J*_1_–*J*_2_ model with small or vanishing *J*_2_ will also exhibit a similar ordering. Finally, note that although the alternative *S* = 1 toy model is useful in the description of elementary states of the block-OSMP system, its validity is limited for the dynamical spin response: it is well known that the dynamical SSF of the *S* = 1 AFM Heisenberg model exhibits “sharp” magnon lines, in contrast to the *S*(*q*, *ω*) of the *S* = 1/2 model that contains a continuum of excitation (at least at low-*ω*), in agreement with our results for the three-orbital Hamiltonian.

### Optical mode

Let us now turn to the high-energy optical mode of the dynamical SSF spectrum. The states contributing to this mode are also triplet excitations. In the *L* = 4 Lanczos analysis, we found that this high-energy mode arises from a state of the form $\left| {\mathrm{O}} \right\rangle _{\gamma = 2}$ ≃ $1{\mathrm{/}}2\left( {\left| { \downarrow \uparrow \uparrow \downarrow } \right\rangle _{\gamma = 2} + \left| { \uparrow \downarrow \downarrow \uparrow } \right\rangle _{\gamma = 2}} \right)$. It is evident that $\left| {\mathrm{O}} \right\rangle$ breaks the FM magnetic unit cells present in the GS. Note, again, that the discussed states do not have doubly occupied or empty sites, reflecting the Mott nature of orbital *γ* = 2. It should be also pointed out that using a small *L* = 4 system with OBC, we have found another state that contributes to the optical mode, i.e., $\left| {\widetilde {\mathrm{O}}} \right\rangle _{\gamma = 2}$ = $1{\mathrm{/}}2\left( {\left| { \downarrow \uparrow \downarrow \uparrow } \right\rangle _{\gamma = 2} + \left| { \uparrow \downarrow \uparrow \downarrow } \right\rangle _{\gamma = 2}} \right)$. However, such a state is not present in the system with periodic boundary conditions.

To understand properly the optical mode it is not enough to focus solely on the localized orbital. A detailed analysis of the remaining “metallic” orbitals *γ* = 0, 1 indicates that: (i) the $\left| {{\mathrm{GS}}} \right\rangle$ and the $\left| {\mathrm{A}} \right\rangle$ states obey the Hund’s rule: spins in different orbitals of the same site are ferromagnetically aligned (see Fig. 4a for a schematic representation). (ii) However, the $\left| {\mathrm{O}} \right\rangle$ states, Fig. 4c, do not fulfill this rule because part of the spins are antiferromagnetically aligned. As a consequence, the main difference in energy between the $\left| {{\mathrm{GS}}} \right\rangle$ and $\left| {\mathrm{O}} \right\rangle$ arises from the local (on-site) Hund exchange portion of the electronic interaction. We confirm this by calculating separately the expectation values of all terms contributing to the Hamiltonian (see Methods section). The main difference between the energy of the $\left| {{\mathrm{GS}}} \right\rangle$ and $\left| {\mathrm{A}} \right\rangle$ arises from the kinetic portion. On the other hand, the difference in $\left| {\mathrm{O}} \right\rangle$ originates, as expected, from the Hund coupling part of the interaction energy. The local on-site nature of the optical mode is also visible in the orbital-resolved SSF. In Fig. 3c, we present the spin correlations between different orbitals at different sites, i.e., *S*_mix_. As clearly visible, the *S*_mix_(*q* → *π*) → 0, indicating a drastic reduction of spectral weight at large momentum. These findings indicate that the optical mode is not present in the inter-orbital inter-site spin correlations. As a consequence, the only remaining possibility of the origin of the optical mode are the intra-site fluctuations between orbitals. Our investigation of orbital-resolved SSF (Fig. 3c) shows that each orbital contributes to the optical mode with a similar weight. Finally, the lack of momentum dependence of the optical mode (at least for *q*/*π* > 1/2) suggests that such excitations are local (on-site) fluctuations of spin between different orbitals at the same site.

**Fig. 6:**
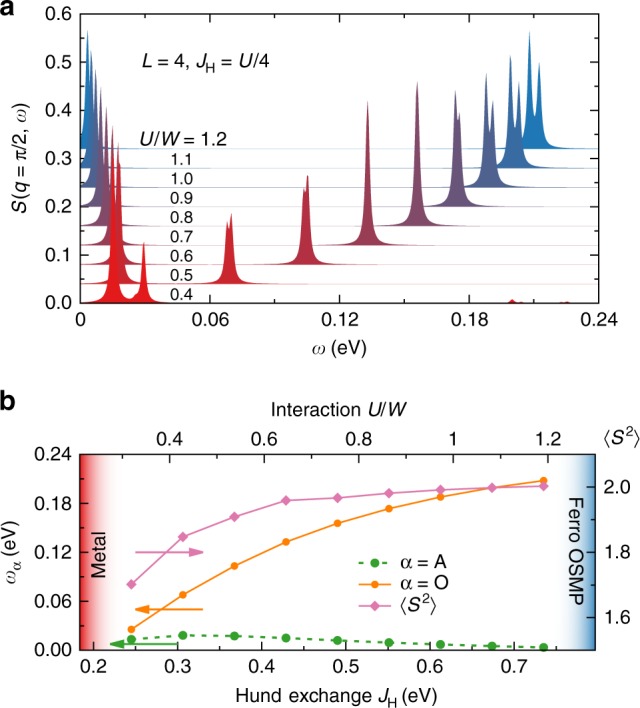
Hund exchange dependence. **a** Dynamical SSF at *q*/*π* = 1/2 for various values of the interaction *U*, all within the block-OSMP phase, *U*/*W* = 0.4, …, 1.2 (bottom to top, with 0.04 offset), at a fixed *J*_H_/*U* = 1/4, calculated for *L* = 4 using the Lanczos method. **b** Left *y*-axis: Frequency *ω*_*α*_ = $\epsilon _\alpha$ − $\epsilon _{{\mathrm{GS}}}$ dependence of the acoustic and optical modes vs the value of the Hund exchange coupling *J*_H_. Right *y*-axis: Magnetic moment $\left\langle {S^2} \right\rangle$ development within the block-OSMP phase

In addition, we have shown that the frequency *ω*_O_ = $\epsilon _{\mathrm{O}}$ − $\epsilon _{{\mathrm{GS}}}$ of the corresponding $\left| {\mathrm{O}} \right\rangle$ excitation is directly proportional to the value of the Hund exchange *J*_H_, contrary to the $\left| {\mathrm{A}} \right\rangle$ excitation with energy $\omega _{\mathrm{A}} = \epsilon _{\mathrm{A}} - \epsilon _{{\mathrm{GS}}}$. In Fig. 6a, we present the dynamical SSF at *q*/*π* = 1/2 for various values of *U* within the block-OSMP calculated via the Lanczos method on *L* = 4 sites, at a fixed *J*_H_/*U* = 1/4. Our results in Fig. 6b indicate that this behavior is valid throughout the entire block-OSMP phase, 0.4 ≲ *U*/*W* ≲ 1.5.

### Ladder geometry

**Fig. 7:**
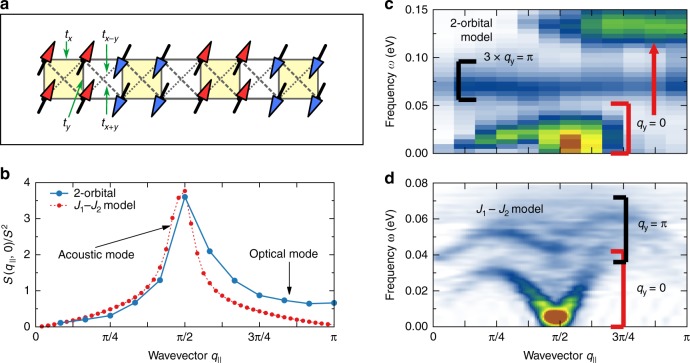
Spin dynamics within the ladder geometry. **a** Schematic representation of the two-orbital two-leg ladder system used in our analysis (see Methods section for details). Arrows depict the 2 × 2 block state. **b** Static SSF in the *q*_*y*_ = 0 sector for both bthe *J*_1_–*J*_2_ and multi-orbital models on a ladder geometry. **c**, **d** Dynamical SSF of the **c** two-orbital and **d** *J*_1_–*J*_2_ spin models on a two-leg ladder geometry (using *L* = 12 and *L* = 48 rungs, respectively)

Finally, let us comment on the lattice geometry dependence of our results. In Fig. 7, we present the SSF for the two-leg ladder two-orbital Hamiltonian introduced in ref.^39^ for the BaFe_2_Se_3_ compound. The lattice is sketched in Fig. 7a and hopping values are given in the Methods section. It was previously shown^39^ that at density $\bar n = 1.75{\mathrm{/}}2$, *J*_H_ = *U*/4, and *U*/*W*_L_ = 2 with *W*_L_ = 3.82 eV the system is in an enlarged block phase, similar to the 2 × 2 block state of BaFe_2_Se_3_^25^. Before addressing specific results, it is important to remark that the DMRG numerical studies of multi-orbital ladders require expensive computations. This is because the inter-site inter-orbital hoppings behave effectively as long-distance hoppings in the equivalent one-dimensional representation, leading to larger entanglement for the ground state (see Supplementary Note 1 for details). The calculation of dynamical quantities is certainly a challenge and even the static expectation values have to be carefully analyzed with regards to the number of states kept (here *M* = 1000 states are used). As a consequence, the results presented for the two-orbital two-leg ladder below may not be as accurate as those for the chains.

On a ladder, there are two separate contributions to the SSF arising from the bonding (*q*_*y*_ = 0) and antibonding (*q*_*y*_ = *π*) sectors. For the two-orbital two-leg ladder results, presented in Fig. 7b, we find a *q*_*y*_ = 0 dispersive mode at low-*ω*, with a continuum of spin excitations similar to the acoustic mode of the chain geometry. At *ω* ≃ 0.075 eV, we find an energy narrow *q*_*y*_ = *π* mode. According to our analysis of the 1D system, a similar spectrum can be found in the *J*_1_–*J*_2_ model on the ladder with FM rung coupling *J*_⊥_ = *J*_1_ (Fig. 7c, d). Both the *J*_1_–*J*_2_ spin model and multi-orbital model on the ladder studied here exhibit the 2 × 2 block state, i.e., AFM-coupled blocks of four FM aligned spins on two neighboring rungs (see Fig. 7a). Such a state has a peak in the static SSF at *q*_*x*_ = *π*/2 in the bonding contribution (*q*_*y*_ = 0), see Fig. 7c. Note that the maximum of the acoustic mode appear at *ω* ≠ 0, which suggests a non-zero spin gap, common in ladders. Finally, at higher frequencies (*ω* ≃ 0.13 eV) in the *q*_*y*_ = 0 sector we find a flat mode of excitations, similar to the optical mode present in the chain analysis. It is again evident that the latter is not captured by the *J*_1_–*J*_2_ model.

## Discussion

Let us compare the INS data for BaFe_2_Se_3_ reported in ref.^25^ against our results. Note that this compound is insulating^22^, while our system Eqs. (1) and (2) for the parameters considered in this work, *U*/*W* = 0.8 and *J*_H_/*U* = 1/4, is a (bad) metal in the block-OSMP phase, becoming insulator only for *U*/*W* ≳ 1.5 in the ferromagnetic-OSMP phase^51^. Our Hamiltonian reproduces the OSMP state and the magnetic block phase of BaFe_2_Se_3_, and although the charge dynamics of our model does not capture the experimentally observed insulating nature of the real material, it is still appealing to study the spin physics. The lack of other multi-orbital models that can reproduce both the spin and charge sector of low-dimensional iron selenides makes it appealing to carry out detailed theoretical calculations of the spin dynamics within this model and compare with the experiments.

Within the spin-wave theory the low-*ω* portion of the INS spectra was interpreted^25^ as a dispersive mode which reflects the frustrated nature of the *π*/2-order. In addition, the high-energy optical modes were interpreted as local excitation of spins within the 2 × 2 plaquette. A similar rationale was used to explain the INS result of the doped compound Rb_0.89_Fe_1.58_Se_2_^35^. The spin-wave theory of BaFe_2_Se_3_ reproduces^25^ all of the modes and also properly captures the frequency bandwidth of the spin excitations. However, only ~2/3 of the total spectral weight expected for localized 3*d* electrons is obtained. Also note that within the considered spin models of refs.^25,35^ unphysically large dimerization spin-exchange couplings are required^52,53^ to stabilize the *π*/2 spin pattern.

From the perspective of our results, the interpretation of the INS spin spectra of low-dimensional ladder iron chalcogenides is different from spin-wave theory. The latter assumes that all excitations occur between localized spins, while in our system we have a mixture of localized and itinerant electrons. Moreover, as shown above, the SSF of multi-orbital systems not only contains dispersive acoustic modes but also local excitations controlled by the Hund exchange, at least within the block-OSMP. The inter-orbital nature of such modes cannot be properly captured by localized Heisenberg models. Our results, on both chain and ladder geometries, indicate that spin models can only properly capture dispersive modes resulting from the peculiar spin order of a given phase as in the *π*/2 state of BaFe_2_Se_3_. However, we argue that only one of the low lying optical modes of this compound arises from a weakly dispersive (probably beyond experimental resolution of powder sample) *q*_*y*_ = *π* excitation. Within our interpretation of the SSF spectra, the second optical mode is of a different nature, involving inter-orbital spin fluctuations on each site. Such a picture is consistent with our multi-orbital ladder results.

Concerning the spectral weight, for the chosen parameters *U*/*W* = 0.8 and *J*_H_/*U* = 1/4 in Eqs.(1) and (2), we observe the magnetic moment $\left\langle {S^2} \right\rangle$ ~ 2 (maximal possible for $\bar n = 4{\mathrm{/}}3$). This is consistent with previous Hartree–Fock calculations^39^ of the block-OSMP phase within a five-orbital ladder system, which reported $\left\langle {S^2} \right\rangle$ ~ 6 for $\bar n = 6{\mathrm{/}}5$ (again the maximal value). As a consequence, our results do not reproduce the missing spectral weight observed in experiments^25^. However, the magnetic moments evolve within the block-OSMP^23^ (see also Supplementary Note 2 for additional results) and only saturate to its maximal value at *U*/*W* ≳ 0.6, namely in the middle of the block-phase. Since the exact value of *U* and *J*_H_ are not know for BaFe_2_Se_3_, it is possible in theoretical investigations to stabilize the block-OSMP phase with a reduced $\left\langle {S^2} \right\rangle$ < 2 (Fig. 6b). Moreover, note that recently it was argued^54^ that insufficient energy (time) resolution in INS experiments produces moments that can be smaller than the actual instantaneous moments. In this context, faster X-ray-based techniques such as photoemission spectroscopy (PES), X-ray absorption spectroscopy (XAS), and X-ray emission spectroscopy (XES) are needed to resolve this issue.

In conclusion, we have investigated the dynamical spin structure factor of a one-dimensional three-orbital Hubbard model in the block orbital-selective Mott phase, as well as a ladder two-orbital Hubbard model also in a similar block state. This has been a computationally demanding effort even with the powerful DMRG, and to our knowledge this is the first time that results of this quality are produced. We have shown that our Hamiltonian captures nontrivial features of a broad family of low-dimensional iron chalcogenides, in particular for the ladder BaFe_2_Se_3_ compound for which *π*/2-block order was reported. We have found two different types of modes in the spin spectra: (i) low-frequency dispersive (acoustic) spin excitations and (ii) optical dispersionless excitations at higher energy. The acoustic band reflects the nature of magnetic order of the system, namely for the block-OSMP the frustrated *π*/2-ordering can be captured by the quantum *J*_1_–*J*_2_ frustrated Heisenberg model, as also shown here. The optical band arises from on-site inter-orbital spin fluctuations controlled by the Hund exchange coupling. Finally, our 1D dynamical SSF is in qualitative agreement with the powder INS spectrum of BaFe_2_Se_3_ (see Supplementary Note 3). Although the latter has only a quasi-1D geometry, with small but non-zero couplings perpendicular to the ladder, the *ω*-dependent spectra should be dominated by the predominantly 1D nature of the system. As a consequence, the location in momentum and energy space is properly resolved by our model Hamiltonian Eqs. (1) and (2) for both of the modes.

Our results are general and should apply to a variety of block states in multi-orbital quasi-1D systems. They should all contain an acoustic band (with pitch wavevector compatible with the size of the magnetic block), a strong asymmetry in the distribution of weight of this acoustic band in different portions of the Brillouin zone, and optical modes with at least one of them related to atomic transitions regulated by the Hund coupling.

## Methods

### DMRG method

The Hamiltonians discussed here were studied using primarily the density-matrix renormalization-group (DMRG) method^55,56^ within the single-center site approach^57^, where the dynamical correlation functions are evaluated via the dynamical DMRG^58–60^, i.e., calculating spectral functions directly in frequency space with the correction-vector method^61^ with Krylov decomposition^60^. The computer package DMRG++ developed at ORNL was used. For a chain geometry, in both stages of the DMRG algorithm, we keep up to *M* = 800 states. This allow us to simulate accurately system sizes up to *L* = 24 sites for dynamical quantities (truncation <10^−8^ for all frequencies *ω*) and *L* = 32 for static quantities (truncation <10^−10^ for the GS). For the ladder geometry results, we use a standard two-site central block approach with *M* = 1000 states (truncation <10^−3^, showing that the two-leg ladder two-orbital results are qualitatively correct, because of its close resemble to the rest, but their quantitative accuracy can be further improved in future efforts). In the Supplementary Note 1, we present the scaling of our results with system size *L*, number of states kept *M*, and broadening *η* of Eq. (5).

### Dynamical SSF

The zero temperature, *T* = 0, total spin structure factor (SSF) *S*(*q*, *ω*) is defined as:

5 $$\begin{array}{ccc}S(q,\omega ) = \frac{1}{\pi }\sqrt {\frac{2}{{L + 1}}} \mathop {\sum}\limits_{\ell = 1}^L {\kern 1pt} {\mathrm{sin}}(q\ell ){\mathrm{sin}}(qL{\mathrm{/}}2) \\ &\times{\mathrm{Im}}{\kern 1pt} \left\langle {{\mathrm{GS}}} \right|\tilde S_\ell \frac{1}{{\omega ^ - - \left( {H - \epsilon _{{\mathrm{GS}}}} \right)}}\tilde S_{L/2}\left| {{\mathrm{GS}}} \right\rangle {\kern 1pt}\end{array} ,$$

with *ω*^−^ = *ω* − *iη*, and $\left| {{\mathrm{GS}}} \right\rangle$ is the ground state with energy $\epsilon _{{\mathrm{GS}}}$. In the above equation, $\tilde S_\ell = \mathop {\sum}\nolimits_\gamma {\kern 1pt} S_{\ell ,\gamma }$ is the total spin on-site $\ell$ for the total *S*SF *S*(*q*, *ω*), or $\tilde S_\ell = S_{\ell ,\gamma }$ for the orbital-resolved SSF $S_{\gamma \gamma \prime }(q,\omega )$.

Furthermore, in the above equation, we adopted the wavevector definition appropriate for open boundary conditions (OBC), i.e., *q* = *kπ*/(*L* + 1) with *k* = 1, …, *L*. As a consequence, in this work we used approximate (exact in the thermodynamic limit *L* → ∞) values of the wave-vectors, e.g., *q* = *π* ≡ *πL*/(*L* + 1).

### Localized basis representation

The eigenstates $\left| \phi \right\rangle$ of the three-orbital system can be written as

6 $$\begin{array}{*{20}{l}} {\left| \phi \right\rangle } \hfill & = \hfill & {\mathop {\sum}\limits_{n = 1}^{64^L} {\kern 1pt} c_n\left| n \right\rangle } \hfill \\ {} \hfill & = \hfill & {\mathop {\sum}\limits_{n_0 = 1}^{4^L} {\kern 1pt} \mathop {\sum}\limits_{n_1 = 1}^{4^L} {\kern 1pt} \mathop {\sum}\limits_{n_2 = 1}^{4^L} {\kern 1pt} c\left( {n_0,n_1,n_2} \right)\left| {n_0} \right\rangle \otimes \left| {n_1} \right\rangle \otimes \left| {n_2} \right\rangle ,} \hfill \end{array}$$

where $\left| n \right\rangle$ represents the orthonormal basis (particle configuration) of all orbitals and $\left| {n_\gamma } \right\rangle$ (with *γ* = 0, 1, 2) represents the particle configuration on given orbital *γ*. Note that $\mathop {\sum}\nolimits_n {\kern 1pt} c_n^2$ = $\mathop {\sum}\nolimits_{n_1,n_2,n_3} {\kern 1pt} c^2\left( {n_1,n_2,n_3} \right)$ = $1$ and $\left\langle {n_\gamma |n_{\gamma \prime }^\prime } \right\rangle = \delta _{nn\prime }\delta _{\gamma \gamma \prime }$. One can rewrite the above equation as

7 $$\left| \phi \right\rangle = \mathop {\sum}\limits_{j = 1}^{4^L} \left| {\tilde c_j} \right\rangle \otimes \left| j \right\rangle _{\gamma = 2},$$

where *j* ≡ *n*_2_ represents—within OSMP—the localized orbital and

8 $$\left| {\tilde c_j} \right\rangle = \mathop {\sum}\limits_{n_0 = 1}^{4^L} {\kern 1pt} \mathop {\sum}\limits_{n_1 = 1}^{4^L} {\kern 1pt} c\left( {n_0,n_1,n_2} \right)\left| {n_0} \right\rangle \otimes \left| {n_1} \right\rangle$$

are vectors. The set of $\left\{ {\left| {\tilde c_j} \right\rangle } \right\}$ vectors represent an orthogonal vector-space with $\mathop {\sum}\nolimits_j \left\langle {\tilde c_j|\tilde c_j} \right\rangle = 1$. Finally, the weight of the $\left| j \right\rangle _{\gamma = {\mathrm{ }}2}$ configuration in the $\left| \phi \right\rangle$ eigenstate is given by the norm of the $\left| {\tilde c_j} \right\rangle$ vector, i.e., $\left\langle {\tilde c_j|\tilde c_j} \right\rangle = \left\| {\tilde c_j} \right\| \equiv \tilde c_j^2$.

### Energy contribution

In Table 1, we present the expectation values of the several terms present in the Hamiltonian Eqs. (1) and (2) for the ground state and also states which contribute to the acoustic and optical modes.

**Table 1:** Energy contributions

	$\epsilon _{\mathrm{k}}$	$\epsilon _{\mathrm{U}}$	$\epsilon _{{\mathrm{U}}\prime }$	$\epsilon _{\mathrm{H}}$	$\epsilon _{\mathrm{P}}$	Total	*ω*_*α*_
$\left| {{\mathrm{GS}}} \right\rangle$	−0.027	8.006	15.280	−1.055	−0.010	22.194	
$\left| {\mathrm{A}} \right\rangle$	0.007	7.993	15.280	−1.065	−0.009	22.206	0.012
$\left| {\mathrm{O}} \right\rangle$	−0.031	8.081	15.262	−0.946	−0.016	22.350	0.156

Kinetic, intra- and inter-orbital interaction, Hund, and pair-hopping energy contributions to the energy of given eigenstates. The last column shows the difference between $\left| {{\mathrm{GS}}} \right\rangle$ and states within the acoustic (red color) and optical (green color) modes. Results are obtained for *L* = 4 and *U*/*W* = 0.8, using the Lanczos method. All numbers in units of eV

### Two-orbital two-leg ladder Hamiltonian

The symmetric hoppings for the two-orbital two-leg ladder system are defined^39^ in orbital space as follows (see sketch in Fig. 7a):

$$\begin{array}{l}t_x = \left( {\begin{array}{*{20}{c}} {0.14769} & 0 \\ 0 & {0.27328} \end{array}} \right),\\ t_y = \left( {\begin{array}{*{20}{c}} {0.28805} & {0.01152} \\ {0.01152} & {0.00581} \end{array}} \right),\\ t_{x \pm y} = \left( {\begin{array}{*{20}{c}} { - 0.21166} & { \mp 0.08430} \\ { \mp 0.08430} & { - 0.18230} \end{array}} \right),\end{array}$$

all expressed in units of eV. The interaction portion of the Hamiltonian is the same as in the 1D system Eq. (2).

### Code availability

Computer codes used in this study are available at https://g1257.github.io/dmrgPlusPlus/.

## Electronic supplementary material

Supplementary Information

## Data Availability

The data that support the findings of this study are available from the corresponding author upon request.
