# Supplementary material for: Spin dynamics of the block orbital-selective Mott phase
Source: Nat Commun. 2018 Sep 13;9:3736. doi: 10.1038/s41467-018-06181-6 (PMC6137186; doi:10.1038/s41467-018-06181-6)
Supplement: Supplementary file 1 — Supplementary Information [file 41467_2018_6181_MOESM1_ESM.pdf]

SUPPLEMENTARY INFORMATION for:

Spin dynamics of the block orbital-selective Mott phase

by J. Herbrych, *et al.*

Supplementary Note 1. Numerical details

In Supplementary Fig. 1 we present the parameter dependence of our dynamical DMRG calculations for a fixed frequency $\omega = 0.03\,[\mathrm{eV}]$ (namely, "inside" the acoustic mode) and $L = 16$ sites (48 orbitals). In panel (a) we present the broadening η dependence of our calculations [Eq. (4) of the main text]. It is clear from the figure that all features are properly resolved for the considered $\eta/\delta\omega = 2$. In Supplementary Fig. 1(b) we present the number of states kept M dependence of our findings. We conclude that at a fixed η and L, the results do not change appreciably for $M \gtrsim 800$.

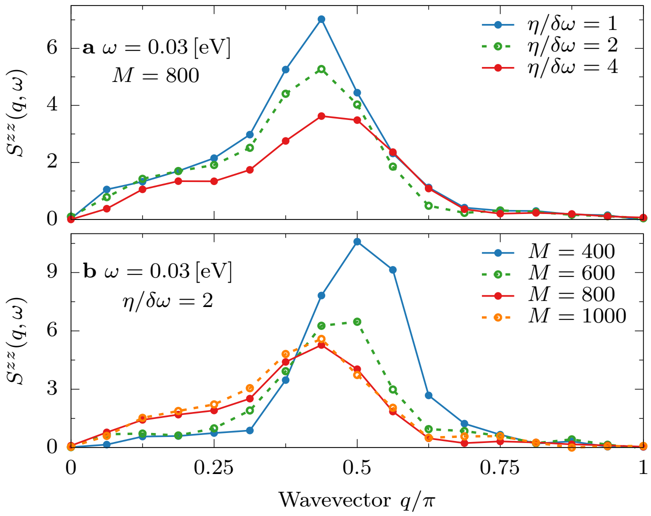

Supplementary Figure 1. **Parameter dependence of dynamical-DMRG simulations.** (a) Broadening η and (b) number of states kept M dependence corresponding to $\omega = 0.03\,[\mathrm{eV}]$ and $L = 16$ sites. In all simulations of the main text we use $\eta/\delta\omega = 2$ and $M = 800$.

In Supplementary Fig. 2(a-d) we present the finite-size analysis at several momenta q cuts through the dynamical SSF. At large $q/\pi \geq 3/4$, the results do not depend on the system size L because for this momentum only the optical mode is present in the spectrum. Since the excitations within this mode are local, the system size (and also dimensionality of the lattice) should not play a crucial role. On the other hand, at $q \leq \pi/2$ the results depend more on the system size with maximal variation at $q/\pi = 1/2$. However, this dependence does not change the main findings of our work and it merely reflects the quasi-long-range nature of the block ordering [1]. This can be understood simply from the L-scaling of the static $S(q = \pi/2)$ shown in the inset of Supplementary Fig. 2(e). For completeness in Supplementary Fig. 2(e) we show the L dependence of the full momentum q resolved static SSF.

Let us finally comment on the accuracy of our results for the multi-orbital ladder geometry. Different from the chain setup, where the three orbitals where treated as a single site with a local Hilbert space of 64 states, the ladder results were obtained using a $12 \times 2 \times 2$ (rungs $\times$ legs $\times$ orbitals) lattice with a local Hilbert space of 4 states. Although such a setup have smaller memory requirements, the entanglement area law [5] heavily influences the accuracy of our results. The latter is a consequence of a large number of long-range connections (up to 7 nearest-neighbours). In Supplementary Fig. 3, we present the system size L and states M scaling of the results presented in Fig. 7 of the main text. In panel (a) we present the finite-size analysis of the static SSF in the bonding sector, $q_y/\pi = 0$, for the $M = 1000$ states kept. The system size analysis of the ladder results is consistent with the findings for chains, namely the acoustic mode has size dependence, while the optical mode does not. In summary, while we are confident that our results for ladders capture the essence of the problem, including the existence of acoustic and optical bands and quite different weights in different portions of the Brillouin zone, only further (very demanding) work can achieve the same accuracy as shown here for chains.

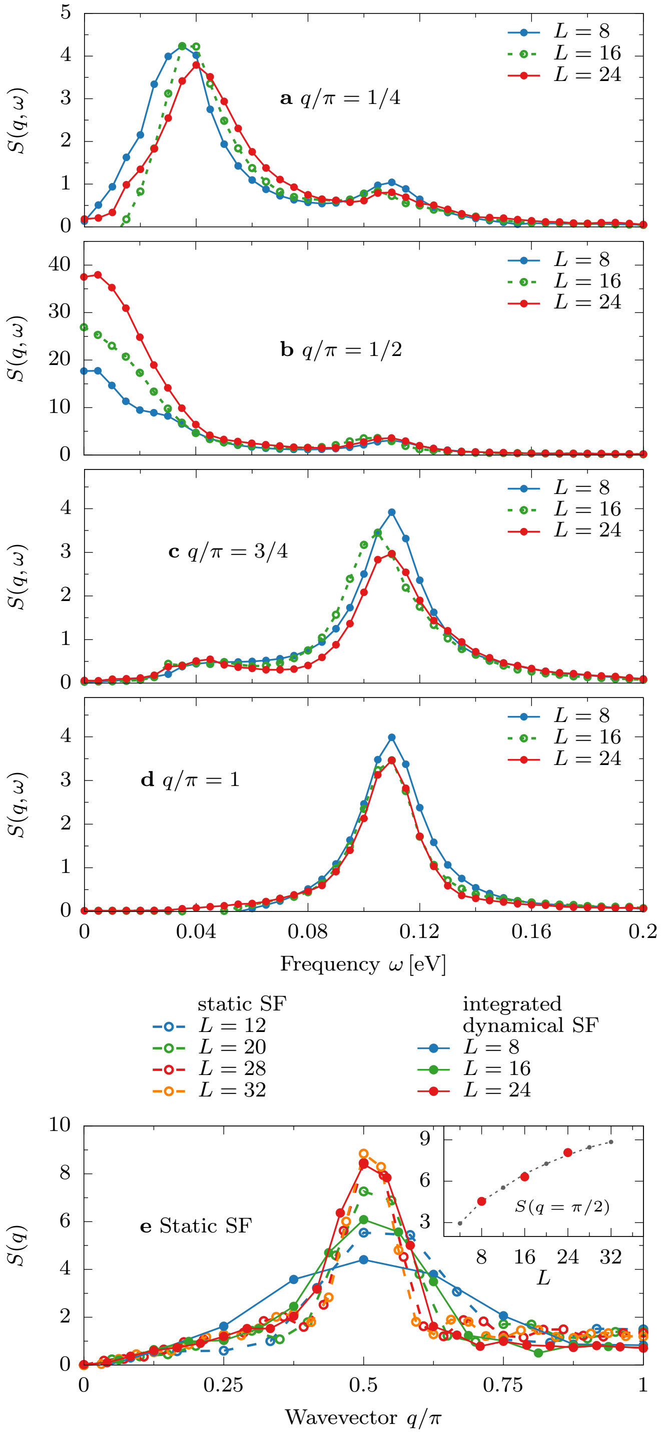

Supplementary Figure 2. **Finite-size analysis.** (a-d) Size L dependence of the frequency-resolved dynamical SSF for $q/\pi = 1/4, 1/2, 3/4, 1$, as calculated with $\eta/\delta\omega = 2$ and $M = 800$. (e) L-dependence of the static SSF. Open points represent the results obtained as the expectation value of the GS, while solid points are obtained from the integral over the frequency (see main text for details). Inset illustrates the quasi-long-range nature of block $\pi/2$ ordering, with a signal intensity growing with L.

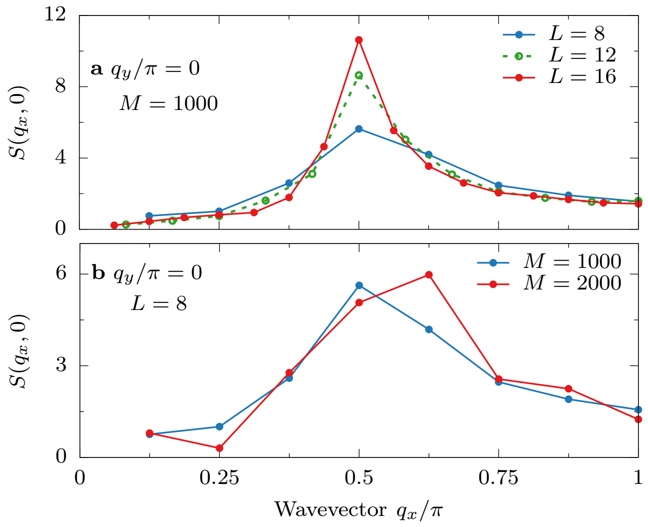

Supplementary Figure 3. **Ladder geometry analysis.** (a) Finite size L and (b) number of states kept M scaling of the static SSF in the bonding sector, $q_y/\pi = 0$.

Supplementary Note 2. Magnetic moment evolution.

In Supplementary Fig. 4 we present the evolution of the local magnetic moment $\langle S^2 \rangle$ within the block-orbital selective Mott phase. This local moment can be obtained from the sum-rules of spin-spin correlation functions, i.e.,

$$S(q) = \frac{1}{\pi} \int \mathrm{d}\omega \, S(q, \omega) \, , \quad \langle S^2 \rangle = \frac{1}{L} \int \mathrm{d}q \, S(q) \, . \tag{1}$$

Note that the above equations allow to relate the total spectral weight of INS data with the value of the local spin via $\langle S^2 \rangle = S(S+1)$. The results presented in Supplementary Fig. 4 are obtained from the integration of the static structure factor $S(q)$. As clearly visible, the magnetic moments start to develop already in the paramagnetic (metallic) phase [1] and are stabilized to its maximal value $\langle S^2 \rangle$ ($S = 1$ for $\overline{n} = 4/3$) in the middle of the block-OSMP.

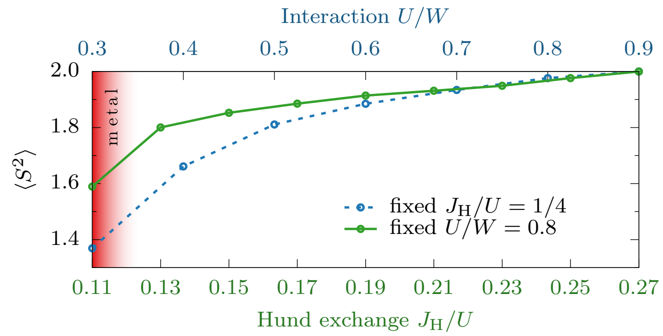

Supplementary Figure 4. **Magnetic moment.** Evolution of the local magnetic moment $\langle S^2 \rangle$ within the block-OSMP. The solid line (lower x-axis) represents results for fixed value of interaction $U/W = 0.8$ and various value of J_H/U. The dashed line (upper x-axis) represents results at fixed $J_\mathrm{H}/U = 1/4$ and for various values of U/W. The results were obtained using a DMRG method with parameters $L = 16$ (48 orbitals), $M = 800$.

Supplementary Note 3. Comparison of DMRG results with powder experiment.

Although $BaFe_2Se_3$ is a quasi-1D compound, the finite ω-dependent properties should be dominated by the 1D nature of the ladder lattice (while, e.g., d.c. transport is more subtle). It is therefore appropriate to directly compare

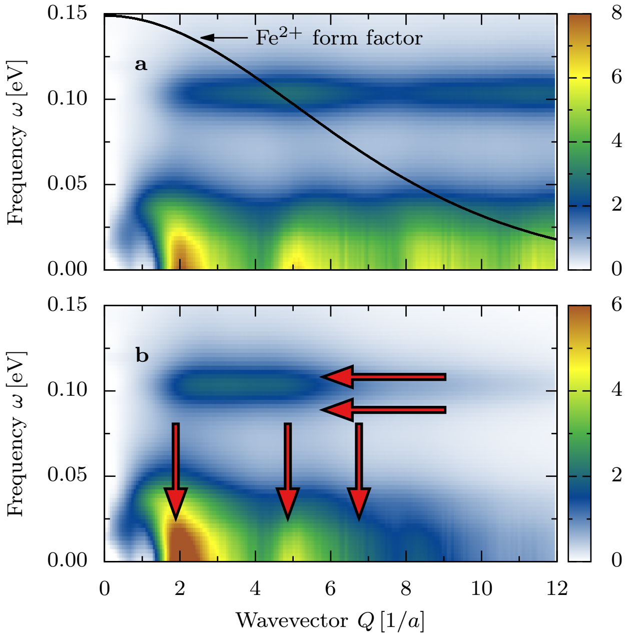

Supplementary Figure 5. **Powder spectrum.** (a) Spherical average of the dynamical SSF. The black solid line represents the magnetic form factor $F(Q)^2$ of the Fe^{2+} ions [3]. (b) Spherical average of the dynamical SSF convoluted with the form factor $F(q)$ relevant for a direct comparison with the $BaFe_2Se_3$ INS results [4]. Red arrows indicate the position of maximum intensities in the INS spectrum. See text for details.

our dynamical SSF to experimental findings. Since the latter is obtained using a powder sample, our results presented in Fig. 2 of the main text have to be averaged over all spherical angles [2]. Furthermore, to qualitatively compare to the inelastic neutron scattering (INS) data we must incorporate in the analysis the momentum dependent magnetic form factors $F(Q)$ of the spin carriers, namely the Fe^{2+} ions. Here we assume a gyromagnetic ratio $g = 2$ (spin-only scattering). The functional form of the former can be taken from crystallography tables [3]. In Supplementary Fig. 5(a) we present the powder average of our spectra. Several interesting general features can be inferred: (i) using realistic values [4] for the Fe-Fe distance such as 2.7 Å, remarkably we obtain a nearly perfect agreement for the position of the acoustic mode. The leading INS signal is centered at $Q \simeq 0.7$ (1/Å), followed by peaks at 1.8 (1/Å) to 2.5 (1/Å) with smaller intensity [indicated by vertical red arrows in Supplementary Fig. 5(b)]. (ii) The neutron spectrum gives three flat (momentum-independent) bands of spin exactions: two of them are centered approximately at $\omega \sim 0.1$ eV ($\omega_1 = 0.0889$ eV and $\omega_2 = 0.1082$ eV, depicted as horizontal red arrows in Supplementary Fig. 5(b)), while the third one is positioned at $\omega_3 = 0.198$ eV. Our 1D results yield only one optical mode centered at $\omega \simeq 0.105$ eV in accord with the most pronounced peak within the INS spectrum. This qualitative agreement indicates that our model is able to capture the nontrivial nature of the frustrated magnetism of $BaFe_2Se_3$, and that the studied parameter range of our Hamiltonian is valid for the whole 123 family.

Supplementary References

[1] J. Rincón, A. Moreo, G. Alvarez, and E. Dagotto, *Exotic Magnetic Order in the Orbital-Selective Mott Regime of Multiorbital Systems*, Phys. Rev. Lett. **112**, 106405 (2014).

[2] K. Tomiyasu, M. Fujita, A. I. Kolesnikov, R. I. Bewley, M. J. Bull, and S. M. Bennington, *Conversion method of powder inelastic scattering data for one-dimensional systems*, Appl. Phys. Lett. **94**, 092502 (2009).

[3] P. J. Brown, Magnetic Form Factors, Chapter 4.4.5, International tables for crystallography vol. C (A. J. C. Wilson, ed.), pp. 391-399.

[4] M. Mourigal, S. Wu, M. B. Stone, J. R. Neilson, J. M. Caron, T. M. McQueen, and C. L. Broholm, *Block Magnetic Excitations in the Orbitally Selective Mott Insulator $BaFe_2Se_3$*, Phys. Rev. Lett. **115**, 047401 (2015).

[5] *Strongly Correlated Systems - Numerical Methods*, edited by A. Avella and F. Mancini (Springer Series in Solid–State Sciences 176, Berlin, 2013).
